# Can effective population size estimates be used to monitor population trends of woodland bats? A case study of *Myotis bechsteinii*

**DOI:** 10.1002/ece3.7143

**Published:** 2021-02-03

**Authors:** Patrick G. R. Wright, Henry Schofield, Fiona Mathews

**Affiliations:** ^1^ Department of Life Sciences University of Sussex Falmer UK; ^2^ Vincent Wildlife Trust Ledbury UK

**Keywords:** Chiroptera, effective population size, genetic monitoring, population monitoring, population trends, wildlife management

## Abstract

Molecular approaches to calculate effective population size estimates (Ne) are increasingly used as an alternative to long‐term demographic monitoring of wildlife populations. However, the complex ecology of most long‐lived species and the consequent uncertainties in model assumptions means that effective population size estimates are often imprecise. Although methods exist to incorporate age structure into Ne estimations for long‐lived species with overlapping generations, they are rarely used owing to the lack of relevant information for most wild populations. Here, we performed a case study on an elusive woodland bat, *Myotis bechsteinii*, to compare the use of the parentage assignment Ne estimator (EPA) with the more commonly used linkage disequilibrium (LD) Ne estimator in detecting long‐term population trends, and assessed the impacts of deploying different overall sample sizes. We used genotypic data from a previously published study, and simulated 48 contrasting demographic scenarios over 150 years using the life history characteristics of this species The LD method strongly outperformed the EPA method. As expected, smaller sample sizes resulted in a reduced ability to detect population trends. Nevertheless, even the smallest sample size tested (*n* = 30) could detect important changes (60%–80% decline) with the LD method. These results demonstrate that genetic approaches can be an effective way to monitor long‐lived species, such as bats, provided that they are undertaken over multiple decades.

## Graphical abstract

Molecular approaches to calculate effective population size estimates (Ne) are increasingly used as an alternative to long‐term demographic monitoring of wildlife populations. Here, we performed a case study on an elusive woodland bat, *Myotis bechsteinii*, to compare the use of the parentage assignment Ne estimator (EPA) (Figure b) with the more commonly used linkage disequilibrium (LD) Ne estimato (Figure a), and assessed the impacts of deploying different overall sample sizes. Our results indicated that the LD method provided more precise estimates (known population size shown in black) and was better suited for the long‐term monitoring of this species.

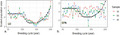

## 1 INTRODUCTION

The implementation of truly long‐term (>100 year) monitoring schemes has been identified as a global priority for wildlife conservation (Frankham, 2010). Yet for many species, even in the absence of political or financial barriers, this is impossible due to the lack of methodologies capable of detecting population trends. Consequently, there has been an increase in the use of effective population size (Ne) estimates, to provide an insight into both demographic and genetic changes in populations (Luikart et al., 2010; Palstra & Fraser, 2012). These molecular approaches along with other indicators of genetic diversity are increasingly being considered to meet global conservation goals (Hoban et al., 2020; IUCN ‐ World Conservation Congress, 2020) and have the potential to supplement or replace traditional long‐term monitoring methods.

Ne is an important parameter because it determines the rate of loss of genetic variability and the rate of increase in inbreeding in a population. Because it is determined both by demographic and genetic processes, it gives an indication of the species' ability to respond to environmental change which cannot be determined from census data alone. For iteroparous species with overlapping generations and/or those with long generation times, the effective number of breeders in a population within a given breeding season (Nb) has been proposed as refinement of Ne and, by including data from a given reproductive bout rather than a generation, is more readily estimated and may give a better short‐term index of a population's current genetic health (Ferchaud et al., 2016; Ruzzante et al., 2016; Waples, 1989, 2005; Whiteley et al., 2017). Because associations are expected between Ne (or Nb) and the censused population size (Nc), genetic markers can potentially be used to assess changes in populations of vulnerable or exploited species (Tallmon et al., 2010). However, the relationships between Ne and Nc are not straightforward (Luikart et al., 2010; Palstra & Fraser, 2012; Pierson et al., 2018). Ne can be defined as the size of an ideal population experiencing genetic drift at the same rate as the observed population (Fisher, 1930; Wright, 1931), but these ideal populations are based on simplified assumptions (e.g., random mating and stable size) that are usually violated in wild populations due to factors such as variable survival, fecundity, or complex mating systems.

Several approaches—using single or multiple sampling—can be applied to estimate Ne. The ease of using a one‐off sampling event means that it is the most widely applied method for assessing populations of wildlife. Techniques to derive Ne from single samples, such as those based on linkage disequilibrium (LD; Hill, 1981; Waples & Do, 2008), molecular coancestry (Nomura, 2008), and excess of heterozygosity (Pudovkin et al., 1996), all provide an assessment of Ne for a given time‐point. However, point estimates of Ne in itself are of limited value for the long‐term monitoring of populations, because i) the relationship with true population size is frequently highly uncertain and ii) conservation management usually requires information on temporal changes in conservation status rather than a single snapshot (Pierson et al., 2018; Schwartz et al., 2007). Temporally spaced genetic sampling schemes have the potential to generate important information for conservation, but examples are generally restricted to commercially valuable species (Bruford et al., 2017).

Most techniques used to calculate Ne, such as the LD method (Hill, 1981; Waples & Do, 2008), assume discrete generations. Provided the interval between sampling events is longer than the interval between generations, the LD method can be used to detect population trends on species exhibiting a iteroparous reproductive strategy (Waples et al., 2014). However, for many long‐lived species, such as mammals, the approach is rarely applied because it would require multiple decades of data (Kamath et al., 2015; Pierson et al., 2018). Long‐term studies where both demographic and genetic materials have regularly been collected for mammals are uncommon, and rarely exceed 10 years. Yet, a study of grizzly bears (*Ursus arctos*) in the Greater Yellowstone Ecosystem has shown that the added information on age structure via long‐term genetic monitoring can help detect population changes over time (Kamath et al., 2015).

Bats, being small, nocturnal, volant, and long‐lived animals that regularly survive more than 20 years in the wild, are particularly hard to monitor using traditional methods (Gaisler et al., 2003). It is potentially possible to obtain reliable population estimates for some species through census counts at accessible roosting sites. However, this is possible for only a minority of species, because many bat species, and particularly tree‐roosting bats, perform fission–fusion behavior (Kerth & Konig, 1999), meaning that census counts are highly unreliable as a significant proportion of the population may be missing. Over half of the world's 1,300+ bat species have unknown population trends (Frick et al., 2019), and so there is considerable interest in the contribution that could be made by genetic monitoring approaches to fill this data gap. For tree‐roosting bats, an even higher proportion of species could potentially benefit, as there is no effective methodology available based on traditional monitoring methods; trends must instead be inferred solely from the state of their habitat.

The Bechstein's bat (*Myotis bechsteinii*) is a woodland specialist, widespread throughout Europe but with a distribution linked to the presence of old growth oak and beech woodlands (Dietz & Pir, 2011; Vergari et al., 1998). Its habitat is thought to have deteriorated sufficiently to permit an inference to be made of a 30% population decline over a 15‐year period, with the expectation that the decline will continue (Paunović, 2016). It is consequently classified as Near Threatened by the IUCN and “in need of strict protection” by the European Habitats Directive (92/43/CEE) (Paunović, 2016).

Until recently, information on the age of individuals was entirely dependent on long‐term banding schemes (Munshi‐South & Wilkinson, 2010). Novel techniques involving the measure of DNA methylation at specific CpG sites have proven to give reliable age estimates of bats, such as *M. bechsteinii* (Wright, Mathews, et al., 2018). Such methods can therefore provide rapid information on the age structure of a population, which could be used to generate more precise calculations of Ne at regular sampling intervals. Here, we applied forward‐time simulations on *M. bechsteinii* populations using life history traits of the species to investigate the potential utility of regular genetic sampling and effective population size estimation over a 150‐year time period as a strategy to monitor population changes. We assessed the efficacy of alternative sampling strategies and compared Ne estimates that incorporated information on the age and sex structure of populations with estimates based on microsatellite data only.

## 2 METHODS

### 2.1 Study sites and sample collection

All *M. bechsteinii* genotypes were obtained from Wright, Hamilton, et al. (2018). This dataset includes genotypes from 260 individuals using 14 microsatellite loci collected at 8 sites. For this study, we only used genotypes from sites in Britain as the simulations were performed on a single British colony/site and there is evidence of genetic structure between mainland Europe and Britain.

### 2.2 Computer simulations

We simulated population changes over a 200‐year period (or breeding cycles) using the forward‐time, individual‐based simulator *simuPOP* (Peng & Amos, 2008; Peng & Kimmel, 2005). Populations from year (breeding cycles) 1 to 50 remained constant and were discarded from further analyses as these were treated as a burn‐in period in order to adjust the provided genotypes with the initial conditions, and then, all genotypes from subsequent years were kept for further analyses. A total of 48 population change scenarios (population decline, expansion, etc.) were created (Supplementary S1). These included 16 single population change scenarios at year 100, and 32 multiple changes scenarios at year 100 and 150 (full details of the scenarios are provided in Supplementary S1). Each simulation provided genotypic outputs along with information on age and sex for all individuals every 5 breeding cycles (years) (this being the generation length of *M. bechsteinii* as defined by the IUCN). Simulations were performed on the population of a single colony/site for which we had 68 genotypes and information on age (Brackett's Coppice, UK; 50.860456–2.6918909) to test for local scale (single colony) population changes.

**Figure 1:**
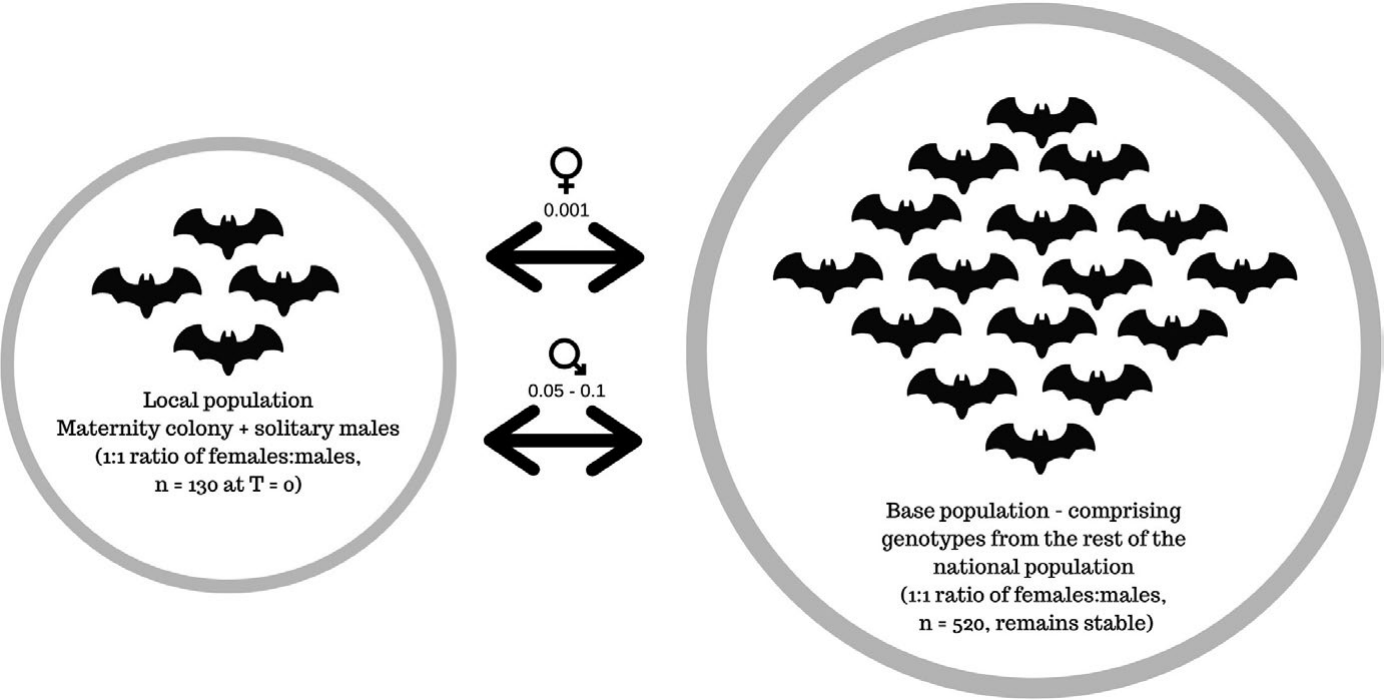
Diagram summarizing the initial settings of the simulations performed on *M. bechsteinii* genotypes. Arrows indicate migration rates

All simulations took into account the life history characteristics of *M. bechsteinii*, such as lifespan, overlapping generations, breeding fitness changes over time and litter size (Supplementary S2). We included a base population comprised of the remaining genotypes from Britain (*n* = 192), which remained constant over time with low levels of migration with the local colony. Although having a closed population may provide more precise estimates, it is biologically unlikely in a species that is known to mix at swarming sites. By accounting for gene flow, the local population would not become isolated from the rest of the base/national population over time. In our simulations, gene flow was adjusted based on expert knowledge and was predominantly governed by males (local population to base population male migration rate = 0.1 and 0.05 the other way), as females live in closed maternity colonies (female migration rate = 0.001). The initial local population size was adjusted at 130 (double the number of female genotypes collected from the maternity colony) with a 1:1 sex ratio to account for both solitary males and the maternity colony and to 520 individuals (double the size of the initial dataset) for the base population (Figure 1).

### 2.3 Effective population size estimates

All effective population size estimates were calculated for the 48 simulations using the 5‐year interval outputs from year 50 to 200 (as the first 50 years were discarded because they were considered a burn‐in period). Estimates were performed using all genotypes of the population for each interval, then 50 and 30 genotypes to account for variation in sample size. Effective population size estimates were first calculated based on the linkage disequilibrium (LD) method using NeEstimator v2, which assumes discrete nonoverlapping generations (Do et al., 2014). A random mating system and a critical threshold for the lowest allele frequency of 0.02 were used for all calculations. As a refinement, we also assessed the impacts of including only individuals from within the same cohort (i.e., reproductive cycle, defined loosely as animals aged 0–3 years, this being the limit of precision for aging the species). This removes the variability otherwise introduced by sampling overlapping generations, and gives an estimate of Nb (Waples et al., 2014). Then, we used the software Age Structure to provide estimates using a parentage assignment estimator (EPA; Wang et al., 2010). Unlike the linkage disequilibrium approach, the EPA method incorporates information on age and sex along with individual genotypes of individuals from a population. All estimates assumed the same life history traits as those included in the simulations, along with a 0.5 probability of including a parent in the dataset.

To clarify, the EPA method uses extra information (age and sex) to provide direct estimates of Ne. In contrast, the LD method, if applied to random samples including a number of consecutive cohorts roughly equal to the generation length, should provide estimates of per‐generation Ne, but these results depend on the sampled age structure (e.g., whether samples are in proportion to the age structure) (Robinson & Moyer, 2013; Waples & Do, 2010). Within single cohorts, the LD method estimates Nb and is expected to apply to a shorter period (3 years), whereas the EPA method estimates the harmonic mean Ne across the generation span prior to the samples being collected (Wang et al., 2010). In our simulations, individuals were sampled randomly and the population age structure did not vary considerably over the simulations, so we expect that the LD estimates will represent a consistent estimate throughout all simulations.

### 2.4 Statistical analyses

The correlation between population size and effective population size estimates, along with confidence intervals, was assessed using normalized cross‐correlations (NCC). Any infinite values were changed to a tenfold increase of the initial population size prior to analysis, to permit them to be included in the final analysis. NCC scores ranged from −1 to 1 (perfect correlation). To assess whether Ne changes were detected immediately or were slightly delayed, we also calculated NCC with time shifts at T1 and T2 (5 and 10 years after sampling, respectively). Correlation scores and confidence interval widths were then compared between both methods with different sample sizes using a two‐way ANOVA. We also calculated Ne/Nc ratios, and standardized population sizes and Ne estimates to a mean of 0 with a standard deviation of 1. Then, we used Bland–Altman plots to assess and compare the precision of Ne estimates for each method with the known population size (Nc).

### 2.5 Detecting population declines

For the method showing the greatest ability to identify population trends, we tested its performance by measuring (a) the ability to detect population declines and (b) the frequency with which a decline was detected in a stable population (false detection). For this, we focused on sudden population declines because the detection of catastrophic events often requires immediate conservation action. We ran 20 simulations of sudden population size declines of 20% occurring at breeding cycle 100. This was repeated for 40%, 60%, and 80% declines. Ne estimates were calculated at 25 years before (Ne‐pre) and 25 years after (Ne‐post) the bottleneck. For each post‐bottleneck Ne (Ne_post_), we calculated whether a decline was successfully detected. A decline was classified as having been detected if Ne_post_ < 0.8 Mean Ne_pre_ (Antao et al., 2011)_._ The false detection of a decline in a stable population was assessed by calculating the mean Ne from all simulations before a bottleneck. This value was used as a reference Ne (Ne_ref_). A false detection was then reported if Ne_pre_ fell under 0.8Ne_ref_.

## 3 RESULTS

**Figure 2:**
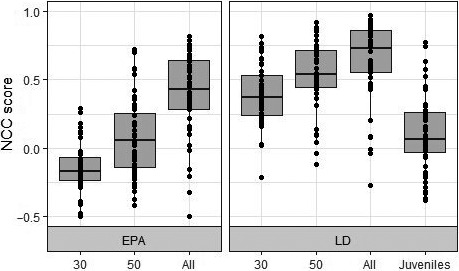
Box plots of normalized cross correlation scores (a score of 1 reflects a perfect correlation) between population size and Ne estimates time series based on the LD and EPA method using different sample sizes for all simulations at a colony

**Figure 3:**
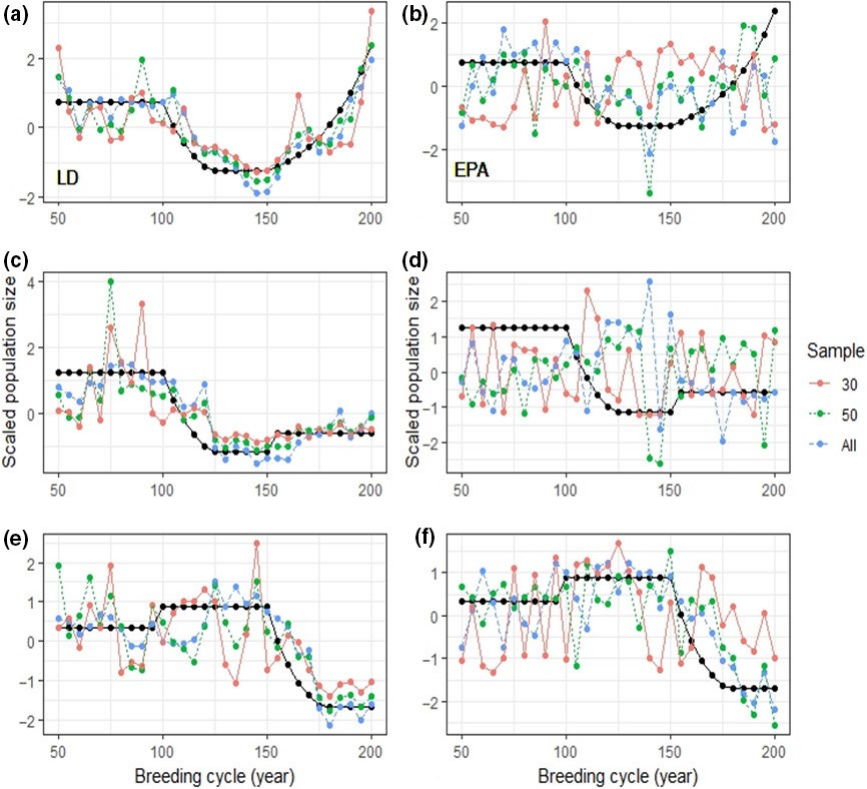
Results from three different simulations (Simulation 21: a, b; Simulation 19: c, b; Simulation 35: e, f) for the LD (left column) and EPA method (right column). The known population size is plotted in black, while estimates using different numbers of samples are in color

We found that NCC scores between true and effective population sizes did not vary according to the timing of sampling (Time Shift *F*_(2,999)_ = 0.535; *p* = .59, 3‐way ANOVA; Supplementary S3). We therefore did not perform further analyses on time periods T1 and T2 as our results suggest that population changes were detected immediately at T0. However, method, sample size, and the interaction between these factors had a significant effect on NCC scores (Method: *F*_(1, 329)_ = 116.97, *p* < .001; Sample size: *F*_(3, 329)_ = 66.282, *p* < .001; Method: Sample size: *F*_(2, 329)_ = 7.763, *p* < .001). Tukey's post hoc test revealed that the LD method performed significantly better than EPA (*p* < .001). All 28 interactions between method and sample size were significant, with the exception of five interactions (Figures 2 and 3). It must also be noted that the NCC scores of five simulations scored under 0.20 for each method tested (Figure 2).

**Figure 4:**
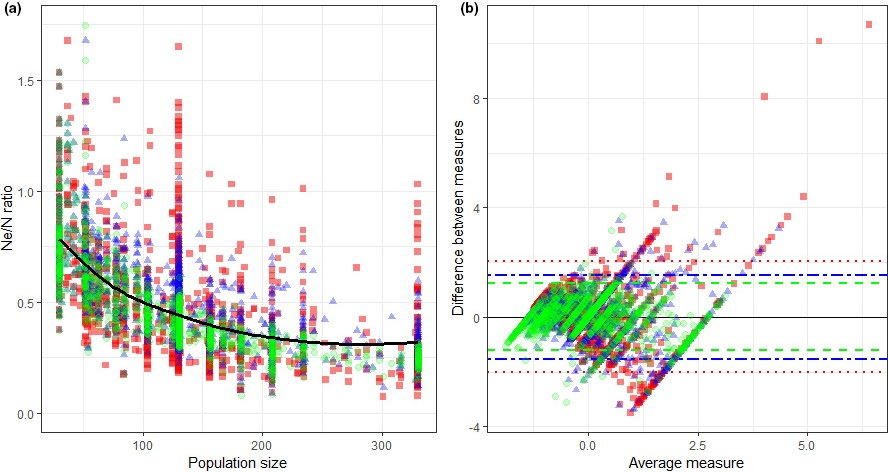
(a) Relationship between Ne/N ratio and population size using the LD method at a local scale with varying sample sizes. (b) Bland–Altman plot with 95% confidence limits of the mean difference using standardized values for N and Ne estimates using the LD method. In both figures, red squares represent a sample size of 30 (CI: dotted), blue triangles 50 samples (CI: two dashed), and green circles (CI: dashed) were used for all samples. A plot evenly scattered above and below zero suggests that there is no consistent bias in the methods used

The relationship between the Ne/Nc ratios and the actual population size was not constant. A log‐linear relationship was identified after a Box–Cox transformation on the Ne/Nc ratios to adjust for heteroscedasticity for LD‐All, LD‐50, and LD‐30 as a result of higher approximation for larger population sizes (LD‐All *R* ^2^ = 0.728, *p* < .001; LD‐50 *R* ^2^ = 0.524, *p* < .001; LD‐30 *R* ^2^ = 0.339, *p* < .001; Supplementary S4, S5, and S6) (Figure 4a). Ne/Nc ratios were higher in smaller populations with a mean Ne/Nc ratio of 0.78 in a population of 30 bats and 0.24 in a population of 330 bats when using all samples for Ne estimates. The Bland–Altman plot confirmed that the precision of the LD method was influenced by the magnitude of the sample size. Differences between Ne and Nc were small when using all samples, but increased when using 50 and 30 samples to calculate Ne and provided better estimates when sampling close to 50% of individuals (Figure 4b and Supplementary S7).

We tested the ability to detect population declines using the LD method as it outperformed the EPA method. Our results showed that 60% and 80% population declines are detected at least 75% of the time after the bottleneck for the following 25 years, with a maximum detection rate at 98.8% when an 80% decline occurs (Table 1 and Supplementary S8 and S9). On the other hand, 20% declines were almost never detected, and 40% declines were detected approximately 50% of the time. The percentage of false declines detected in a stable population 25 years prior to the bottleneck was similar when analyses used either all samples (17.6%) or 50 samples (17.4%), but increased when using 30 samples (29.5%).

**Table 1:** Percentage Ne estimates detecting a significant population decline after a bottleneck at breeding cycle 100

Decline	Percentage Ne estimates detecting a decline		
	All samples	50 samples	30 samples
20%	13 (16.3)	25 (28.8)	27 (27.5)
40%	53 (62.5)	54 (63.8)	55 (62.5)
60%	66 (80)	74 (88.8)	66 (75)
80%	85 (95)	89 (98.8)	86 (96.3)

Note

Here, we classified a decline as significant if Ne_post_ < 0.80 × average Ne_pre_ (Percentages in brackets were calculated by omitting Ne estimates at breeding cycle 100).

## 4 DISCUSSION

Genetic monitoring has the potential to provide valuable information on the population trends of elusive species. While such methods are commonly applied in fisheries (e.g., Christie et al., 2012), they are rarely used with long‐lived wild mammals and birds. Recent advances in molecular aging techniques have provided the opportunity to test whether models incorporating additional information on population age and sex structure would improve the precision of Ne estimates. By using genotypic data and forward‐time simulations, we were able to compare the ability of alternative sampling strategies to detect population trends of a rare bat species, *M. bechsteinii*.

Despite including additional information on population age and sex structure, the EPA method (Wang et al., 2010), was strongly outperformed by the more commonly used LD method when using the same sample size. While the single‐sample LD method has been used and tested on numerous species (e.g., Murphy et al., 2018; Waples et al., 2018), the EPA method developed by Wang et al. (2010) has not received as much attention. Work on grizzly bears by Kamath et al. (2015) in Yellowstone indicated that despite providing similar results as other methods, the EPA was more sensitive to a decrease in sample size. Sampling schemes that favor the collection of closely related individuals may cause underestimates; and errors in age estimates may lead to increased variability (Wang et al., 2010). For the study of bats, such limitations may be important drawbacks as most samples per site are likely to originate from a maternity colony where all individuals are somewhat related, and age estimates are likely to be approximate. Furthermore, the requirement of additional information on populations (e.g., sampling proportions of age classes and sex) that is rarely available from wild populations may further reduce the precision of the Ne estimates as such information is not always available. The LD method, on the other hand, solely requires genotypes and is therefore less subject to such errors despite lacking any extra information on sex and age of individuals.

The importance of sample size for the LD method in detecting population declines was previously highlighted by Antao et al. (2011) through simulations, and in empirical work (Kamath et al., 2015). Our results show that increasing sample size clearly improved the capacity to detect population trends. Yet, large declines (60% – 80%) could still be detected using the LD method despite reducing sample size. For small declines, Waples and Do (2010) suggested that increasing the number of loci would have a similar effect as increased sample size. Single nucleotide polymorphisms (SNPs) provide a promising alternative to traditionally used microsatellites and many hundreds of SNPS can be readily identified across the genome. Yet, Antao et al. (2011), on the other hand, found that increased sample sizes are far better suited to detect rapid population declines.

In the case of age‐structured populations, the grouping of several consecutive cohorts using the single‐sample LD method can provide robust estimates of Ne, representative of the number of breeders (Nb) (Waples et al., 2014). We therefore assessed the impacts of repeatedly sampling only bats aged 0–3 years old. However, our results indicate that estimates using this approach performed similarly to the LD method using 30 samples from individuals of all ages. Reduced precision using this method may be directly linked to variations in sample size as when bottlenecks occur, the number of juveniles is often reduced to very few individuals in a colony. Robinson and Moyer (2013) have previously reported that sampling only juveniles gives accurate estimates of Nb, but the best estimates of Ne are derived from sampling across the reproductively active population, particularly where reproductive success is skewed toward older age groups (as is the case with bats).

The Ne/Nc ratio is important for understanding the risk that demographic, environmental, and genetic factors have on the viability of populations, because Ne is usually smaller than the true population size (Palstra & Fraser, 2012). Yet, this relationship can be hard to assess as it can be affected by either habitat factors or population changes over time (Belmar‐Lucero et al., 2012; Fraser et al., 2007). Although the high ratios observed are likely to be a result of the sensitivity of the Ne estimation methods to small sample sizes, our work suggests that Ne/Nc ratios ranging from 0.24 to 0.78 are plausible for slow breeding mammals (Hoban et al., 2020), but there is little evidence to permit comparison of these results with other bat species. When using the LD method, we found that Ne/Nc ratios showed a log‐linear relationship with N, which agrees with Palstra and Fraser (2012). In wild mammal populations, the short duration of most studies means that such trends remain unclear. For example, Kamath et al. (2015) found that Ne/Nc ratios remained constant while Pierson et al. (2018) highlighted the difficulties in finding any consistent trends over time. It is therefore generally recommended that Ne is used primarily as a metric to detect changes over time as opposed to assessing population size (Pierson et al., 2018). For IUCN red list assessments, this means that genetic data at present could only contribute toward criterion A (population size reduction based on Ne) and will not provide information on criterion C (small population size and decline) or D (very small or restricted population) as these depend on estimates of the known number of mature adults (IUCN, 2012).

### 4.1 Conservation implications/applications

Appropriate planning of wildlife monitoring schemes is vital if they are to be robust and cost‐efficient. Here, we summarize essential points that must be considered for the setup of a genetic monitoring program for *M. bechsteinii,* or any other woodland bat.

- The LD method appears more robust than the EPA method. Although age structure may not be essential in the calculation of Ne, molecular aging techniques still have an important place in population monitoring as they may help in the detection of small declines (e.g., high proportion of old individuals) and enable estimates of Nb to be made for each cohort.
- Long‐term monitoring with large sampling intervals (~ 5 years) should be prioritized. The detection of any trend in Ne for a long‐lived species with overlapping generations requires a long time series where sampling interval should be similar to the generation length of the species (R. S. Waples, personal communication, 2018). For this study, we used a sampling interval equivalent to the generation length of *M. bechsteinii* as defined by the IUCN.
- Sample size is a primary factor in determining the power of a monitoring program to detect population trends. The use of 30 samples per colony may, at times, be insufficient to provide precise estimates, with better results being obtained when close to half of the population was sampled. We recommend that a minimum of 30–50 samples should be collected for tree‐roosting bats because colonies rarely exceed 100 individuals. This number can be obtained by prioritizing sites where bats commonly use boxes and by including juveniles in the sampling program. Higher numbers of samples, however, may be needed for maternity colonies of species roosting in high numbers (>1,000 bats) in caves.

## 5 CONCLUSIONS

This study uses pre‐existing genotypic data and life history characteristics of an elusive long‐lived woodland bat, *M. bechsteinii*, to test the use of genetic monitoring techniques and inform best practice for the long‐term monitoring of this species. Methods to incorporate age structure in Ne estimates do exist, but are rarely used as such information on wild populations is scarce. With the development of molecular aging techniques, Ne estimators may be more commonly used on iteroparous species but information on their precision is still lacking. By simulating a multitude of demographic scenarios, and testing different Ne estimate methods using variable sample sizes, we identified the main limitations and provided recommendations on their application for *M. bechsteinii*. The EPA method, which takes into account age and sex, performed poorly, because it also requires additional information on the population that is not always available (e.g., probability of sampling a parent). The LD method, however, performed well and, in this study, is better suited for detecting population trends over time, if sample size is large enough. This study, using simulations over long periods of time, is the first to test the possibility of monitoring woodland bat population trends using molecular approaches and offers insights into the most appropriate sampling strategy.

## CONFLICT OF INTEREST

The authors report no conflict of interest.

## AUTHOR CONTRIBUTIONS

**Patrick G. R. Wright:** Conceptualization (lead); formal analysis (lead); methodology (equal). **Henry Schofield:** Conceptualization (equal); funding acquisition (lead); supervision (lead); writing‐review & editing (equal). **Fiona Mathews:** Conceptualization (equal); funding acquisition (lead); supervision (lead); writing‐review & editing (lead).

## Supporting information

Supplementary Material

## Data Availability

All necessary data for this study are available on the Figshare digital repository: https://figshare.com/s/b83627773d92ca6ab8a3.
