# Supplementary material for: Can effective population size estimates be used to monitor population trends of woodland bats? A case study of Myotis bechsteinii
Source: Ecol Evol. 2021 Feb 3;11(5):2015–23. doi: 10.1002/ece3.7143 (PMC7920762; doi:10.1002/ece3.7143)
Supplement: Supplementary file 1 — Supplementary Material [file ECE3-11-2015-s001.docx]

**Supplementary material**

Supplementary 1: Summary of all demographic scenarios performed.

Supplementary 2: Summary of settings used simulate *Myotis bechsteinii* life history.

| Setting | Value |
| --- | --- |
| Maximum lifespan | 17 |
| Minimum breeding age | 2 |
| Maximum breeding age | 17 |
| Number of young per year | 1 |
| Mating | Random |
| Fertility | Decreases with age |


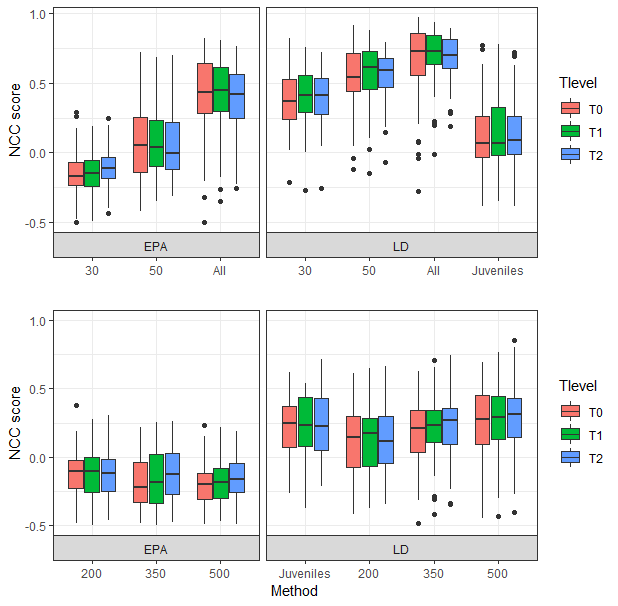


Supplementary 3: Comparison of normalised cross correlation scores between Ne at T0, T1 (5 years after sampling) and T2 (10 years after sampling) to test for any lag in detecting population changes and population size time series for all simulations at a colony and national level.

**
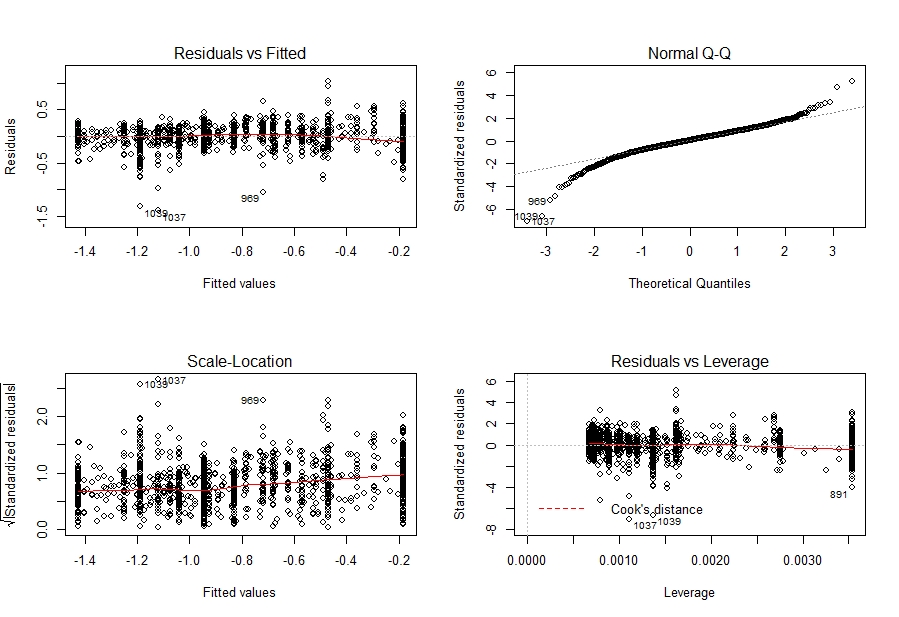
**

Supplementary 4: Model plots testing the relationship Ne/N ratios and population size using all samples

**
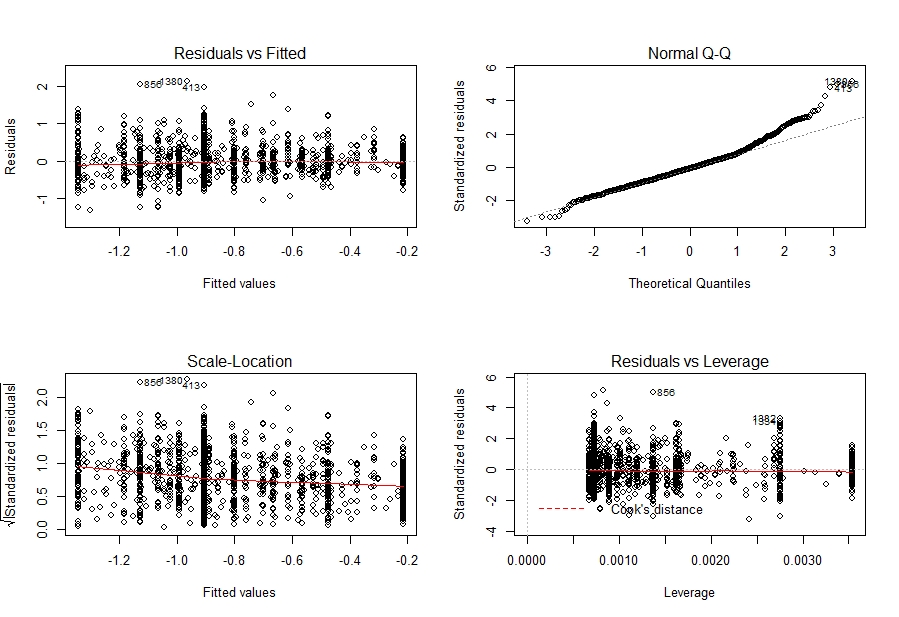
**

Supplementary 5: Model plots testing the relationship Ne/N ratios and population size using 50 samples.

**
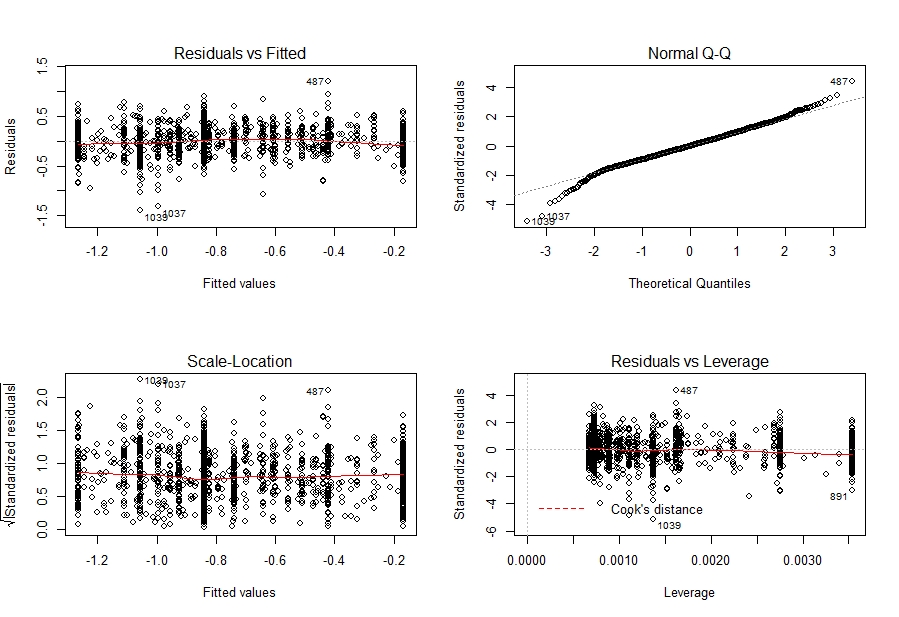
**

Supplementary 6: Model plots testing the relationship Ne/N ratios and population size using 30 samples.


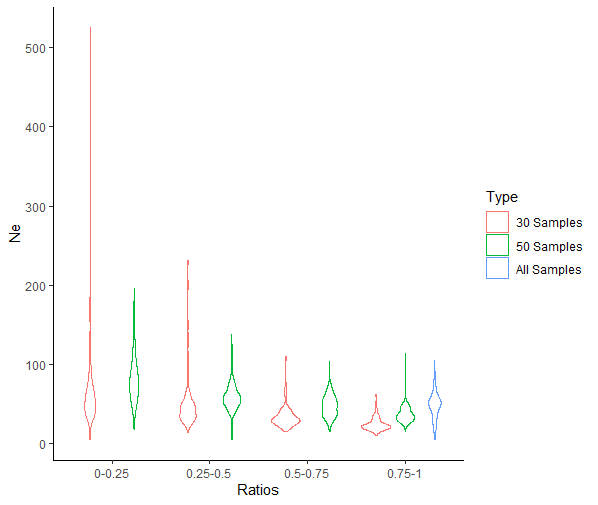


Supplementary 7: Violin plots representing the variation in LD-Ne estimates with the sample size/Nc ratios when using 30, 50 and all samples.

Supplementary 8: Mean Ne estimates 25 years pre and post-bottleneck (Ne estimates in brackets were calculated by omitting Ne estimates at breeding cycle 100).

|  | Ne (All samples) | | Ne (50 samples) | | Ne (30 samples) | |
| --- | --- | --- | --- | --- | --- | --- |
| Decline | Pre | Post | Pre | Post | Pre | Post |
| 20% | 50.66 | 48.01 (47.21) | 55.55 | 53.93 (53.43) | 51.78 | 57.26 (58.04) |
| 40% | 53.53 | 43.23 (41.27) | 59.81 | 48.31 (45.32) | 58.23 | 48.84 (45.73) |
| 60% | 52.18 | 39.17 (35.36) | 60.48 | 41.85 (37.73) | 58.98 | 42.51 (38.23) |
| 80% | 51.97 | 30.63 (26.10) | 60.087 | 30.63 (26.10) | 60.65 | 32.25 (28.13) |


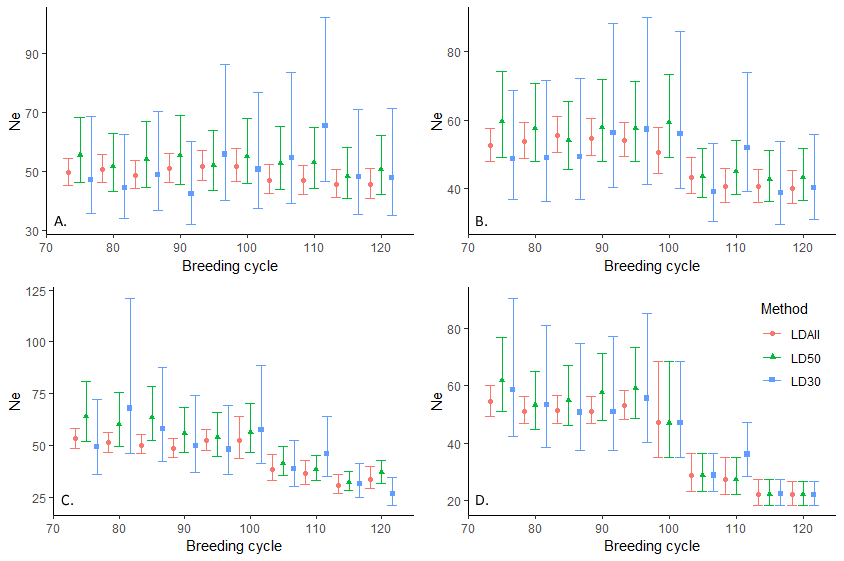


Supplementary 9: Median Ne estimate, upper and lower confidence intervals of all replicates after undergoing at breeding cycle 100 a (A) 20% decline; (B) 40% decline; (C) 60% decline and (D) an 80% decline.
